# A Newton-like iterative method implemented in the DelPhi for solving the nonlinear Poisson-Boltzmann equation

**DOI:** 10.3934/mbe.2020331

**Published:** 2020-09-21

**Authors:** Chuan Li, Mark McGowan, Emil Alexov, Shan Zhao

**Affiliations:** 1 Department of Mathematics, West Chester University of Pennsylvania, West Chester, Pennsylvania, 19383, USA; 2 Department of Mathematics, University of Alabama, Tuscaloosa, AL 35487, USA; 3 Department of Physics and Astronomy, Clemson University, Clemson, South Carolina, 29634, USA

**Keywords:** DelPhi, Poisson-Boltzmann equation, electrostatics, Newton method, Finite difference technique

## Abstract

DelPhi is a popular scientific program which numerically solves the Poisson-Boltzmann equation (PBE) for electrostatic potentials and energies of biomolecules immersed in water via finite difference method. It is well known for its accuracy, reliability, flexibility, and efficiency. In this work, a new edition of DelPhi that uses a novel Newton-like method to solve the nonlinear PBE, in addition to the already implemented Successive Over Relaxation (SOR) algorithm, is introduced. Our tests on various examples have shown that this new method is superior to the SOR method in terms of stability when solving the nonlinear PBE, being able to converge even for problems involving very strong nonlinearity.

## 1. Introduction

Electrostatic interaction is a major factor which is commonly taken into account when studying numerous biological phenomena[1,2], such as macromolecular binding and recognition[3–6], pH-dependent folding and binding[7–11], nonspecific ion binding[12–14], pKa calculations[15–17], and salt-dependent effects[18,19], etc.. Existing models of calculating electrostatic potentials and corresponding energies developed in the past couple of decades can be roughly classified into two categories. Explicit solvent models treat mobile water and ions explicitly and thus capture all molecular details but are computationally costly in terms of CPU time and memory usage. Implicit solvent models, such as the Generalized Born[20] and Poisson-Boltzmann (PB) models[21–26], treat surrounding water as a continuum media, and can be solved with relatively low computational costs. Because of that, implicit solvent models are usually preferred when modeling electrostatics of macromolecules at genome-scale applications.

Among all existing implicit solvent models, the Poisson-Boltzmann equation (PBE), is one of the most popular models utilized by many researchers. A lot of efforts have been devoted to developing scientific software to solve the PBE. For instance, DelPhi[27,28] utilizes the finite difference and Successive Over Relaxation (SOR) methods to iteratively solve the PBE until a prescribed tolerance is satisfied, PBSA[29] adopts the Finite Volume/Periodic Conjugate Gradient (FV/PCG) and the Immersed Interface/Fast Fourier Transform (IIM/FFT) methods to solve the PBE, MIBPB[30] develops a unique matched interface and boundary (MIB) method to explicitly enforce the jump conditions on the interfaces (molecular surfaces) in the finite difference formulations, resulting in a method capable of capturing sharp jumps of the potentials at the molecular surfaces, APBS[31] is an adaptive PBE solver which solves the PBE by a specifically designed finite element method, and many others[32,33].

As one of the most popular PBE solvers, DelPhi has been continuously maintained and developed for improved performance. Many new features were added in DelPhi in recent years[34]. This work reports a newly developed Newton-like method which was introduced into DelPhi recently. This new method has been tested extensively, including some purposely created “crashing” cases with strong nonlinearity. In particular, this method has been shown to be incredibly stable and is capable of delivering reliable numerical results in all tested cases, making this newly developed method a valuable add-on to DelPhi for solving problems with strong nonlinearity.

The rest of this work is organized as follows. The PBE and the finite difference methods are presented in section 2. Benchmarks of selected examples are shown section 3 to numerically compare the two methods implemented in DelPhi, followed by Conclusions and Acknowledgements in section 4 and 5, respectively.

## 2. Methods

### 2.1. The Poisson-Boltzmann Equation (PBE)

The PBE[35] is an elliptic-type Partial Differential Equation (PDE) given by

(1) $$\nabla {}^{\circ}\cdot \left(\varepsilon \left(x\right)\nabla \phi \left(x\right)\right)-\kappa {\left(x\right)}^{2}\sinh\left(\phi \left(x\right)\right)=\;-4\pi \rho \left(x\right),$$

where $\phi \left(x\right)$ is the electrostatic potential, $\varepsilon \left(x\right)$ is a spatial dielectric function, $\kappa \left(x\right)$ is a modified Debye-Huckel parameter, and $\rho \left(x\right)$ is the charge distribution function. Eqn. (1) is usually referred to as the Nonlinear Poisson-Boltzmann Equation (NLPBE) due to the presence of the hyperbolic sine function, $\sinh\left(\phi \left(x\right)\right)$, in eqn. (1). If the potential $\phi \left(x\right)$ is known to be small, eqn. (1) can be linearized by an approximation, $\sinh\left(\phi \left(x\right)\right)\approx \phi \left(x\right)$, yielding a simplified model

(2) $$\nabla {}^{\circ}\cdot \left(\varepsilon \left(x\right)\nabla \phi \left(x\right)\right)-\kappa {\left(x\right)}^{2}\phi \left(x\right)=\;-4\pi \rho \left(x\right),$$

commonly referred to as the Linearized Poisson-Boltzmann Equation (LPBE). It is known that exact solutions to eqn. (1) – (2) only exist for a few simplified cases[27]. In practice, they must be solved numerically via certain numerical treatments for real bio-objects due to their irregular shapes. The numerical approaches for handling the nonlinearity of the PBE can be classified into two categories. In the most commonly used approach, the PBE is discretized by using finite difference or finite element methods, resulting a nonlinear algebraic system. Then a nonlinear algebraic method, such as nonlinear relaxation method [36,37], nonlinear conjugate gradient method [38] or inexact Newton method [39], can be employed to solve the nonlinear system efficiently. A comprehensive assessment of various algebra-based nonlinear PBE solvers can be found in [40]. A pseudo-time approach has also been developed [41–43], in which a time-dependent PBE is introduced by adding a pseudo-time derivative, and the PBE solution is recovered by a steady-state integration. The pseudo-time approach is usually less efficient than the nonlinear algebraic approach, because a long-time integration is needed for the steady state. But the pseudo-time approach could be more stable, especially when an analytical treatment to the nonlinear term is applied [43]. The method proposed in this work belongs to the first category. In the following subsections, implemented numerical methods in the DelPhi program will be introduced.

### 2.2. The Successive Over Relaxation (SOR) method

DelPhi solves eqn. (1) – (2) in a cubic domain Ω containing the interested molecule. Boundary conditions are imposed on the six faces of Ω. Domain Ω is discretized by a uniform mesh size h=Δx=Δy=Δz in all *x*-, *y*-, and *z*- directions. Approximations to the exact solutions of eqn. (1) – (2) are to be found at all grids.

**Figure 1.**
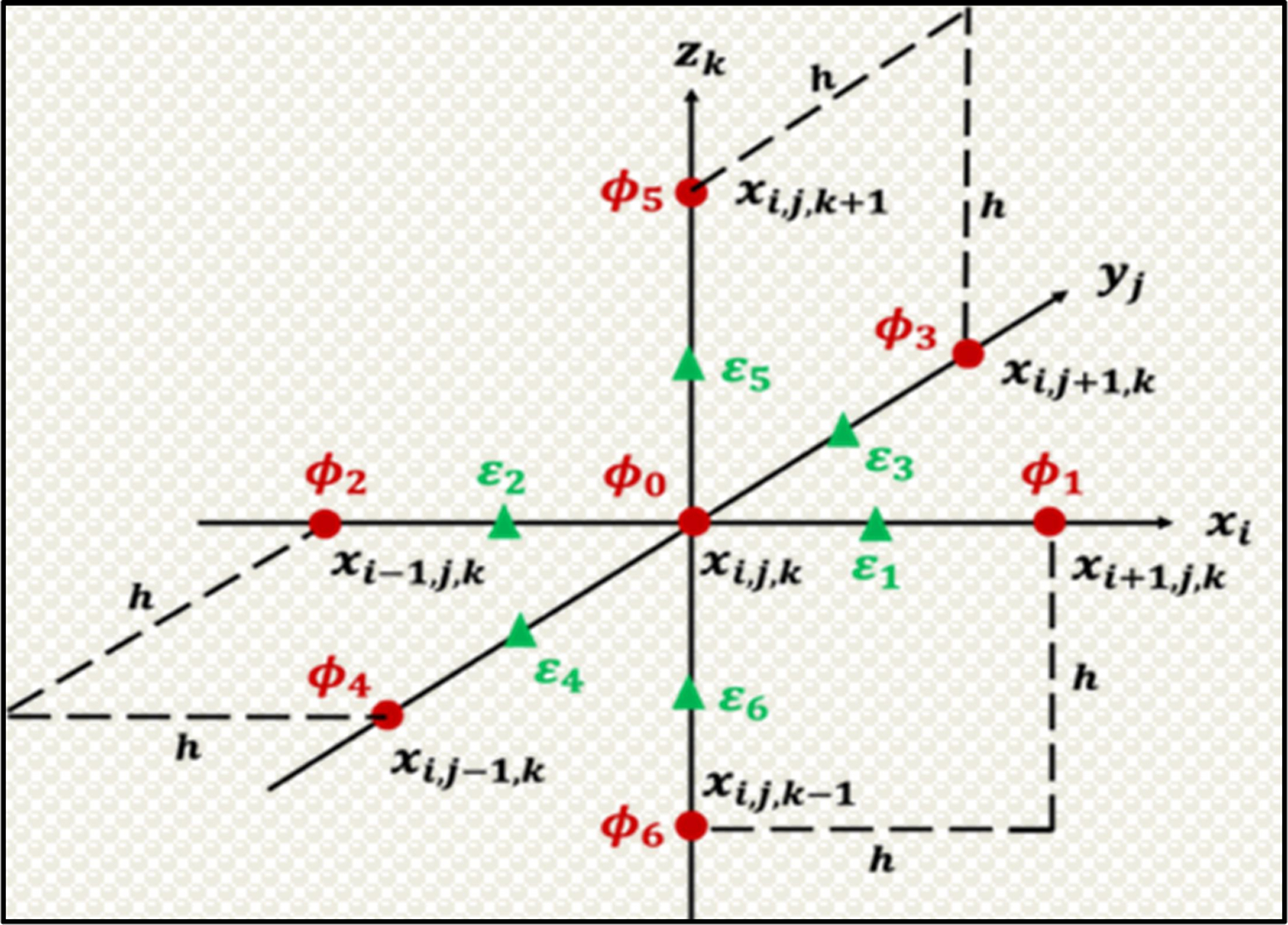
A graph demonstration of the numerical methods implemented in the DelPhi solver.

Following the standard finite difference formulation, eqn. (1) is discretized, resulting in[27]

(3) $$h\sum_{i=1}^{6}\varepsilon_{i}\phi_{i}-h\sum_{i=1}^{6}\varepsilon_{i}\phi_{0}-\kappa^{2}\sinh\left(\phi_{0}\right)h^{3}+4\pi q_{0}=0,$$

where $\phi_{0}$ is the unknown potential at a grid $x_{i,j,k}$, $q_{0}$ is the charge assigned to the same grid $x_{i,j,k}$, $\phi_{i},i=1\ldots 6$ are unknown potentials at six closest adjacent grids, and $\varepsilon_{i},i=1\ldots 6$ are dielectric coefficients at six adjacent half grids. See Figure 1 for a demonstration. Eqn. (3) can be rewritten as an iteration updating formula

(4) $$h\sum_{i=1}^{6}\varepsilon_{i}\phi_{i}^{n}-h\sum_{i=1}^{6}\varepsilon_{i}\phi_{0}^{n+1}-\kappa^{2}\sinh\left(\phi_{0}^{n}\right)h^{3}\frac{\phi_{0}^{n+1}}{\phi_{0}^{n}}+4\pi q_{0}=0$$

with the superscript n=0,1,… indicating the number of iterations. Solving $\phi_{0}^{n+1}$ in terms of others in eqn. (4) yields [35]

(5) $$\phi_{0}^{n+1}=\left(\sum_{i=1}^{6}\varepsilon_{i}\phi_{i}^{n}+\frac{4\pi q_{0}}{h}\right)/\left(\sum_{i=1}^{6}\varepsilon_{i}+{\left(\kappa h\right)}^{2}\frac{\sinh\left(\phi_{0}^{n}\right)}{\phi_{0}^{n}}\right)$$

to solve the NLPBE for the potential at grid $x_{i,j,k}$. In a similar fashion, one can obtain the formula[44]

(6) $$\phi_{0}^{n+1}=\left(\sum_{i=1}^{6}\varepsilon_{i}\phi_{i}^{n}+\frac{4\pi q_{0}}{h}\right)/\left(\sum_{i=1}^{6}\varepsilon_{i}+{\left(\kappa h\right)}^{2}\right)$$

to solve the LPBE for the potential at $x_{i,j,k}$. Provided a guessed value of $\phi_{0}^{0}$ (usually called the initial value), the current (approximated) potential $\phi_{i}^{n}$ is evolved to the next (approximated) potential $\phi_{0}^{n+1}$ by either eqn. (5) or (6) in the *n*th step of an iteration process for n=1,2,…. This process is terminated until a prescribed criterion is satisfied.

In DelPhi, potentials $\phi_{0}^{n+1}$ and $\phi_{0}^{n}$ in eqn. (5) – (6) are used in the SOR method

(7) $$\phi_{0}^{n+1}=\omega \phi_{0}^{n+1}+\left(1-\omega \right)\phi_{0}^{n}$$

for improved efficiency or stability in the nth iteration as well. Here the relaxation parameter ω is selected to be 0<ω<2. When a value 0<ω<1 is used, the iteration process converges slower but more stably (under relaxation). When a value 1<ω<2 is used, the iteration process converges in a faster pace but could be less stable (over relaxation). DelPhi uses ω=1 as the default value, yielding a method commonly known as the Gauss-Seidel (GS) method. DelPhi users can either manually set the value of ω, or let the program automatically calculate the optimized values of ω, for either faster convergence rates or stronger stability.

Eqn. (5) provides a numerical formula to solve the NLPBE iteratively but its convergence rate is not fast enough to solve problems in three dimensions[44]. DelPhi utilizes a special technique to accelerate the convergent rate. To this end, eqn. (1) is “linearized” and rewritten as

(8) $$\nabla {}^{\circ}\cdot \left(\varepsilon \left(x\right)\nabla \phi \left(x\right)\right)-\kappa {\left(x\right)}^{2}\phi \left(x\right)=\;-4\pi \rho \left(x\right)+\kappa {\left(x\right)}^{2}\left(\sinh\left(\phi \left(x\right)\right)-\phi \left(x\right)\right),$$

where the nonlinear term, $\kappa {\left(x\right)}^{2}\left(\sinh\left(\phi \left(x\right)\right)-\phi \left(x\right)\right)$, acts as an “excess charge” added to the regular charge term on the right-hand side of eqn. (8)[44]. When eqn. (8) is discretized, a formula

(9) $$\phi_{0}^{n+1}=\left(\sum_{i=1}^{6}\varepsilon_{i}\phi_{i}^{n}+\frac{4\pi q_{0}}{h}-\frac{\chi \left(\kappa {\left(x\right)}^{2}\left(\sinh\left(\phi_{0}^{n}\right)-\phi_{0}^{n}\right)\right)}{h}\right)/\left(\sum_{i=1}^{6}\varepsilon_{i}+{\left(\kappa h\right)}^{2}\right)$$

is derived in place of eqn. (5) for solving the NLPBE. In eqn. (9), the excess charge term is multiplied by a second relaxation (strength) parameter χ which is initially small, χ=0.05. This parameter is slowly increased as iteration moves forward until χ=1 is reached. Then “full” nonlinear iterations start with χ=1 along the way.

In DelPhi, eqn. (6) and (7) are coupled for solving the LPBE, and eqn. (7) and (9) are coupled for solving the NLPBE. The iteration process is terminated, for instance, when $\left|\phi_{0}^{n+1}-\phi_{0}^{n}\right|<\text{TOL}$ at all grids for a prescribed tolerance TOL. These methods, together with additional computational techniques, such as the “checkerboard” ordering, stripping, and contiguous memory mapping^35^, have been proven to be able to effectively deliver accurate numerical solutions to the LPBE and NLPBE for many three-dimensional problems.

However, it is known that the aforementioned “excess charge” treatment is merely a computational technique which could lead to undesired divergences caused by potentials at grids in water passing certain threshold, the grid spacing, and other factors [44]. One such “bizarre” example in which the SOR method fails to converge is given in the next section. It calls for a new addition to DelPhi’s capabilities, namely a Newton-like method, primarily focusing on solving the NLPBE for problems with strong nonlinearity. This method is described in the next subsection.

### 2.3. A Newton-like (NWT) method

The NWT method was developed to improve the stability of the numerical procedure when solving the NLPBE for problems with strong nonlinearity. To this end, we reconsider the left-hand side of eqn. (3) as a function of $\phi_{0}$ and write

(10) $$F\left(\phi_{0}\right)=h\sum_{i=1}^{6}\varepsilon_{i}\phi_{i}-h\sum_{i=1}^{6}\varepsilon_{i}\phi_{0}-\kappa^{2}\sinh\left(\phi_{0}\right)h^{3}+4\pi q_{0}.$$

In order to find the root(s) of the equation $F\left(\phi_{0}\right)=0$ via the Newton’s algorithm, the derivate of $F\left(\phi_{0}\right)$ is calculated first

(11) $$\frac{dF}{d\phi_{0}}=-h\sum_{i=1}^{6}\varepsilon_{i}-h^{3}\kappa^{2}\cosh\left(\phi_{0}\right).$$

Then eqn. (10) – (11) are substituted in the Newton’s algorithm, yielding

(12) $$\begin{array}{l} \phi_{0}^{n+1}=\;\;\;\;\;\;\;\;\;\;\;\;\;\;\;\;\;\;\;\;\;\;\;\;\;\;\;\;\;\;\;\;\;\;\;\;\phi_{0}^{n}-\frac{F\left(\phi_{0}^{n}\right)}{\frac{dF}{d\phi_{0}}\left(\phi_{0}^{n}\right)}\\ \;\;\;\;\;\;\;\;⬚=\phi_{0}^{n}-\frac{h\sum_{i=1}^{6}\varepsilon_{i}\phi_{i}^{n}-h\sum_{i=1}^{6}\varepsilon_{i}\phi_{0}^{n}-\kappa^{2}\sinh\left(\phi_{0}^{n}\right)h^{3}+4\pi q_{0}}{-h\sum_{i=1}^{6}\varepsilon_{i}-h^{3}\kappa^{2}\cosh\left(\phi_{0}^{n}\right)}\\ \;\;\;\;\;\;\;\;⬚=\frac{\sum_{i=1}^{6}\varepsilon_{i}\phi_{i}^{n}+\frac{4\pi q_{0}}{h}+{\left(\kappa h\right)}^{2}\left(\phi_{0}^{n}\cosh\left(\phi_{0}^{n}\right)-\sinh\left(\phi_{0}^{n}\right)\right)}{\sum_{i=1}^{6}\varepsilon_{i}+{\left(\kappa h\right)}^{2}\cosh\left(\phi_{0}^{n}\right)}. \end{array}$$

Eqn. (12) can be treated as a new updating formula to evolve $\phi_{0}^{n}$ to $\phi_{0}^{n+1}$. Moreover, one can see that there is no difficulty to couple eqn. (7) and (12) and embrace all techniques already implemented in DelPhi to solve the NPBE.

Following similar derivations, eqn. (2) can be discretized as

(13) $$h\sum_{i=1}^{6}\varepsilon_{i}\phi_{i}-h\sum_{i=1}^{6}\varepsilon_{i}\phi_{0}-\kappa^{2}h^{3}\phi_{0}+4\pi q_{0}=0.$$

Defining

(14) $$G\left(\phi_{0}\right)=h\sum_{i=1}^{6}\varepsilon_{i}\phi_{i}-h\sum_{i=1}^{6}\varepsilon_{i}\phi_{0}-\kappa^{2}h^{3}\phi_{0}+4\pi q_{0},$$

one can calculate $G^{\prime }\left(\phi_{0}\right)$ as

(15) $$\frac{dG}{d\phi_{0}}=-h\sum_{i=1}^{6}\varepsilon_{i}-h^{3}\kappa^{2}.$$

Substituting eqn. (14) – (15) in the Newton’s algorithm yields

(16) $$\phi_{0}^{n+1}=\;\;\;\;\;\;\;\;\;\;\;\;\;\;\;\;\;\;\;\;\;\;\;\;\;\;\;\;\;\;\;\;\;\;\phi_{0}^{n}-\frac{G\left(\phi_{0}^{n}\right)}{\frac{dG}{d\phi_{0}}\left(\phi_{0}^{n}\right)}\;\;\;\;\;\;\;\;\;⬚=\phi_{0}^{n}-\frac{h\sum_{i=1}^{6}\varepsilon_{i}\phi_{i}^{n}-h\sum_{i=1}^{6}\varepsilon_{i}\phi_{0}^{n}-\kappa^{2}h^{3}\phi_{0}^{n}+4\pi q_{0}}{-h\sum_{i=1}^{6}\varepsilon_{i}-h^{3}\kappa^{2}}\;\;\;\;\;\;\;\;\;⬚=\;\;\;\;\;\;\;\;\;\;\;\;\;\;\;\;\;\;\;\;\;\;\;\;\;\;\;\;\;\;\;\frac{\sum_{i=1}^{6}\varepsilon_{i}\phi_{i}^{n}+\frac{4\pi q_{0}}{h}}{\sum_{i=1}^{6}\varepsilon_{i}+{\left(\kappa h\right)}^{2}}$$

for solving the LPBE.

Eqn. (16) is actually the same as eqn. (6). That is, both SOR and NWT methods utilize the same numerical formula to solve the LPBE. Thus, it is expected that these two methods are equally accurate and efficient for solving the LPBE. An example is provided in the supplementary material to numerically verify that implementations of these two methods in DelPhi are indeed equally accurate and efficient. Therefore, we will concentrate on comparing their performance when different formulas are actually used to solve the NLPBE in the remaining of this work.

The novelty of the NWT method is two-fold. First, eqn. (12) is derived by applying the Newton algorithm on discretized equations obtained from the original PBE, while other Newton-type PBE solvers in the literature, to our best knowledge, are obtained by applying the Newton’s algorithm directly on the original PBE. Secondly, this NWT method is implemented in a way to inherit all unique computational techniques, except the “excess charge”, already implemented in the DelPhi solver. One can view this new NWT method as a DelPhi-specialized Newton-like method which is not seen elsewhere.

### 2.4. Comparison

Three iteration formulas, eqn. (5), (9), and (12), have been presented in this section for solving the NLPBE. It will be interesting to compare them side by side and provide our understanding of these iteration formulas. In order to simplify the discussions, we assume that a mesh size *h* is fixed and h≪1. We focus on just one iteration step, the nth iteration, in which the potential $\phi_{0}^{n}$ at an arbitrary grid is evolved to $\phi_{0}^{n+1}$ by one of these three formulas. Moreover, we assume all potentials on the right-hand side of three equations all take on the same values in the nth iteration step. Noticing that the three formulas become identical at grids inside the molecule/protein because the modified Debye-Huckel parameter $\kappa \left(x\right)=0$ in this case. Therefore, performance differences can only be observed at grids immersed in water. We thus limit our analysis to the solvent domain, where the potential function is smooth and bounded because no point charges locate there. Thus, in the water, it is reasonable to assume $\phi_{i}^{n}\approx \phi_{0}^{n},i=1,\ldots ,6$ in these formulas for a small but fixed *h*. When being stable, these three formulas will converge to the same solution as *n* goes to infinity. Such a solution will be called the algebraic solution, which satisfies the finite difference discretization of the NLPBE, i.e., eqn. (3).

We will investigate these three formulas in two aspects, i.e., compare their convergence rates and analyze their stabilities when the potential is large. Eqn. (5) is considered first. When $\phi_{0}^{n}$ is small, eqn. (5) is reduced to eqn. (6) by approximating $\sinh\left(\phi_{0}^{n}\right)\approx \phi_{0}^{n}$. In addition, by the assumption of $\phi_{i}^{n}\approx \phi_{0}^{n},i=1,\ldots ,6$, eqn. (5) can be viewed as a linear function, $\phi_{0}^{n+1}\approx a\phi_{0}^{n}+b$ for some constants *a* and *b*, where 0<a<1. Thus, $\phi_{0}^{n+1}$ converges in a linear rate with respect to $\phi_{0}^{n}$. When $\phi_{0}^{n}$ is large but still on track, the right-hand side of eqn. (5) has a much large denominator than that of eqn. (6) because $\frac{\sinh\left(\phi_{0}^{n}\right)}{\phi_{0}^{n}}\gg 1$. This drives eqn. (6) to converge to the NLPBE potential. Nevertheless, when $\phi_{0}^{n}$ is large and away from the limiting value, stability has to be analyzed. Assuming $\phi_{i}^{n}\approx \phi_{0}^{n}$ and neglecting constants, the dominate term of eqn. (5) can be expressed as $C{\left(\phi_{0}^{n}\right)}^{2}/\sinh\left(\phi_{0}^{n}\right)$ for some constant *C*. Because the denominator is much larger than the numerator, this iteration will not blow up and thus remains stable. In total, we view the series of potentials ${\left\{\phi_{0}^{n+1}\right\}}_{n=0,1,2,\ldots }$ calculated by eqn. (5) are stably converging to the algebraic solution of eqn. (3). However, it is known that ${\left\{\phi_{0}^{n+1}\right\}}_{n=0,1,2,\ldots }$ converges not quickly enough for solving three-dimensional problems[44].

The SOR method utilizing eqn. (9) aims at making the iteration process converge in a faster pace. To see this, we rewrite eqn. (9) as

(17) $$\begin{array}{l} \phi_{0}^{n+1}=\left(\sum_{i=1}^{6}\varepsilon_{i}\phi_{i}^{n}+\frac{4\pi q_{0}}{h}-\frac{\chi \left(\kappa {\left(x\right)}^{2}\left(\sinh\left(\phi_{0}^{n}\right)-\phi_{0}^{n}\right)\right)}{h}\right)/\left(\sum_{i=1}^{6}\varepsilon_{i}+{\left(\kappa h\right)}^{2}\right)\\ \;\;\;\;\;\;\;\;⬚=\;\;\;\;\;\;\;\;\;\;\;\;\;\;\frac{\sum_{i=1}^{6}\varepsilon_{i}\phi_{i}^{n}+\frac{4\pi q_{0}}{h}}{\sum_{i=1}^{6}\varepsilon_{i}+{\left(\kappa h\right)}^{2}}-\frac{\chi \left(\kappa {\left(x\right)}^{2}\left(\sinh\left(\phi_{0}^{n}\right)-\phi_{0}^{n}\right)\right)/h}{\sum_{i=1}^{6}\varepsilon_{i}+{\left(\kappa h\right)}^{2}}, \end{array}$$

where the first term on the right-hand side is the same as the right-hand side of eqn. (6), and the second term can be viewed as a “correction” added to the first term for improved convergence rate. When $\phi_{0}^{n}$ is small, $\sinh\left(\phi_{0}^{n}\right)\approx \phi_{0}^{n}$ so that the correction term does not contribute much to $\phi_{0}^{n+1}$. In this case eqn. (9) converges in a similar rate as that of eqn. (5) and (6). When $\phi_{0}^{n}$ is large, $\sinh\left(\phi_{0}^{n}\right)-\phi_{0}^{n}\gg 1$ and $\left(\sinh\left(\phi_{0}^{n}\right)-\phi_{0}^{n}\right)/h$ is even larger provided h≪1 so that the correction term becomes a significant portion in $\phi_{0}^{n+1}$ and drives $\phi_{0}^{n+1}$ in an accelerated pace towards the algebraic solution of the discretized NLPBE. However, the correction term could also introduce additional issues. When $\phi_{0}^{n}\gg 1$, the value of $\left(\sinh\left(\phi_{0}^{n}\right)-\phi_{0}^{n}\right)/h$ could drive $\phi_{0}^{n+1}$ stride to be overshot the solution. Assuming $\phi_{i}^{n}\approx \phi_{0}^{n}$ and neglecting constants, the dominate term of eqn. (6) takes a form of $a\;sinh(\phi_{0}^{n})-b\phi_{0}^{n}$ for some constants *a* and *b*. Consequently, the potential could grow exponentially, and the whole iteration process quickly diverges. DelPhi utilizes a couple of relaxations parameters, ω in eqn. (7) and χ in eqn. (9), in order to pull $\phi_{0}^{n+1}$ back to the range of the solution in the overshot situation. These relaxation techniques work in most situations, but there is no guarantee that they are always effective. For instance, one “crashing” example is demonstrated in the next section that the SOR method faces severe difficulties to converge.

Eqn. (9) has been proven to cope with most cases in practice and it has other advantages over eqn. (5). First of all, it allows the same computational and programming techniques flawlessly shared between solving the LPBE and NLPBE. Secondly, the denominator on the right-hand side of eqn. (9) is unchanged in all iterations so that it can be calculated once, saved and then reused in all iterations. It is very computationally economical. Third, eqn. (9) can collaborate with other advanced techniques in DelPhi, resulting in one of the best PBE solvers in the world. Overall, we believe the SOR method implemented in DelPhi is an effective method to solve the NLPBE for three-dimensional problems.

The newly developed NWT method utilizes eqn. (12) in order to maintain stability when solving the NLPBE for problems with strong nonlinearity, while it is still able to converge in a rate faster than the method using eqn. (5). To see this, we rewrite eqn. (12) as

(18) $$\begin{array}{l} \phi_{0}^{n+1}=\;\;\;\;\;\;\frac{\sum_{i=1}^{6}\varepsilon_{i}\phi_{i}^{n}+\frac{4\pi q_{0}}{h}+{\left(\kappa h\right)}^{2}\left(\phi_{0}^{n}\cosh\left(\phi_{0}^{n}\right)-\sinh\left(\phi_{0}^{n}\right)\right)}{\sum_{i=1}^{6}\varepsilon_{i}+{\left(\kappa h\right)}^{2}\cosh\left(\phi_{0}^{n}\right)}\\ \;\;\;\;\;\;\;\;⬚=\frac{\sum_{i=1}^{6}\varepsilon_{i}\phi_{i}^{n}+\frac{4\pi q_{0}}{h}}{\sum_{i=1}^{6}\varepsilon_{i}+{\left(\kappa h\right)}^{2}\cosh\left(\phi_{0}^{n}\right)}+\frac{{\left(\kappa h\right)}^{2}\left(\phi_{0}^{n}\cosh\left(\phi_{0}^{n}\right)-\sinh\left(\phi_{0}^{n}\right)\right)}{\sum_{i=1}^{6}\varepsilon_{i}+{\left(\kappa h\right)}^{2}\cosh\left(\phi_{0}^{n}\right)}\\ \;\;\;\;\;\;\;\;⬚=\;\;\;\;\;\;\;\frac{\sum_{i=1}^{6}\varepsilon_{i}\phi_{i}^{n}+\frac{4\pi q_{0}}{h}}{\sum_{i=1}^{6}\varepsilon_{i}+{\left(\kappa h\right)}^{2}\cosh\left(\phi_{0}^{n}\right)}+\frac{{\left(\kappa h\right)}^{2}\left(\phi_{0}^{n}-\tanh\left(\phi_{0}^{n}\right)\right)}{\sum_{i=1}^{6}\varepsilon_{i}/\cosh\left(\phi_{0}^{n}\right)+{\left(\kappa h\right)}^{2}}, \end{array}$$

where the first term on the right-hand side is similar to that of eqn. (17) with one additional $\cosh\left(\phi_{0}^{n}\right)$ in the denominator, and the second term, which is still called the correction term, is new in the NWT method. When $\phi_{0}^{n}$ is small, the first term is practically the same as that of eqn. (17) because $\cosh\left(\phi_{0}^{n}\right)\approx 1$, and the second term vanishes because $\tanh\left(\phi_{0}^{n}\right)\approx \phi_{0}^{n}$. In this case eqn. (12) converges in a similar rate as that of eqn. (5), (6) and (9). When $\phi_{0}^{n}$ s large but still on track, $\left|\sinh\left(\phi_{0}^{n}\right)\right|\approx \cosh\left(\phi_{0}^{n}\right)\gg \left|\phi_{0}^{n}\right|>1$ so that the first term on the right-hand side of eqn. (18) is smaller than the right-hand side of eqn. (5). Together with the second term, this will drive the potential convergent to the algebraic solution of the discretized NLPBE in a faster pace than that of eqn. (5). When $\phi_{0}^{n}$ is large and far apart from the algebraic solution, the dominate term of eqn. (12) behaves like $a\phi_{0}^{n}+b\tanh\left(\phi_{0}^{n}\right)+c\phi_{0}^{n}/\cosh\left(\phi_{0}^{n}\right)$ for some constants *a*, *b*, and *c* by assuming $\phi_{i}^{n}\approx \phi_{0}^{n}$ and neglecting constants. This iteration only grows linearly as $\phi_{0}^{n}$ increases. This is essentially why the NWT method is more stable than the SOR method.

In summary, we believe that eqn. (5) could provide a stable method to solve the NLPBE. However, its relatively low convergence rate makes it unsuitable to solve the NLPBE for three-dimensional problems. The SOR method improves the convergence rate by an “exponential” correction term. This correction term allows the iterations progress in a fast pace, but it could lead to unexpected divergence for problems with high nonlinearity. The NWT method substitutes the correction term with a moderate one to balance the needs for both efficiency and stability, and we expect it to be a useful alternative of the SOR method in DelPhi to solve problems with high nonlinearity.

## 3. Results

Benchmarks are presented to compare the SOR and NWT methods in this section. Both methods have been implemented in DelPhi using the same computational and programing techniques. A wide selection of examples was tested, and three examples are chosen to demonstrate due to the limited length of this work.

**Figure 2.**
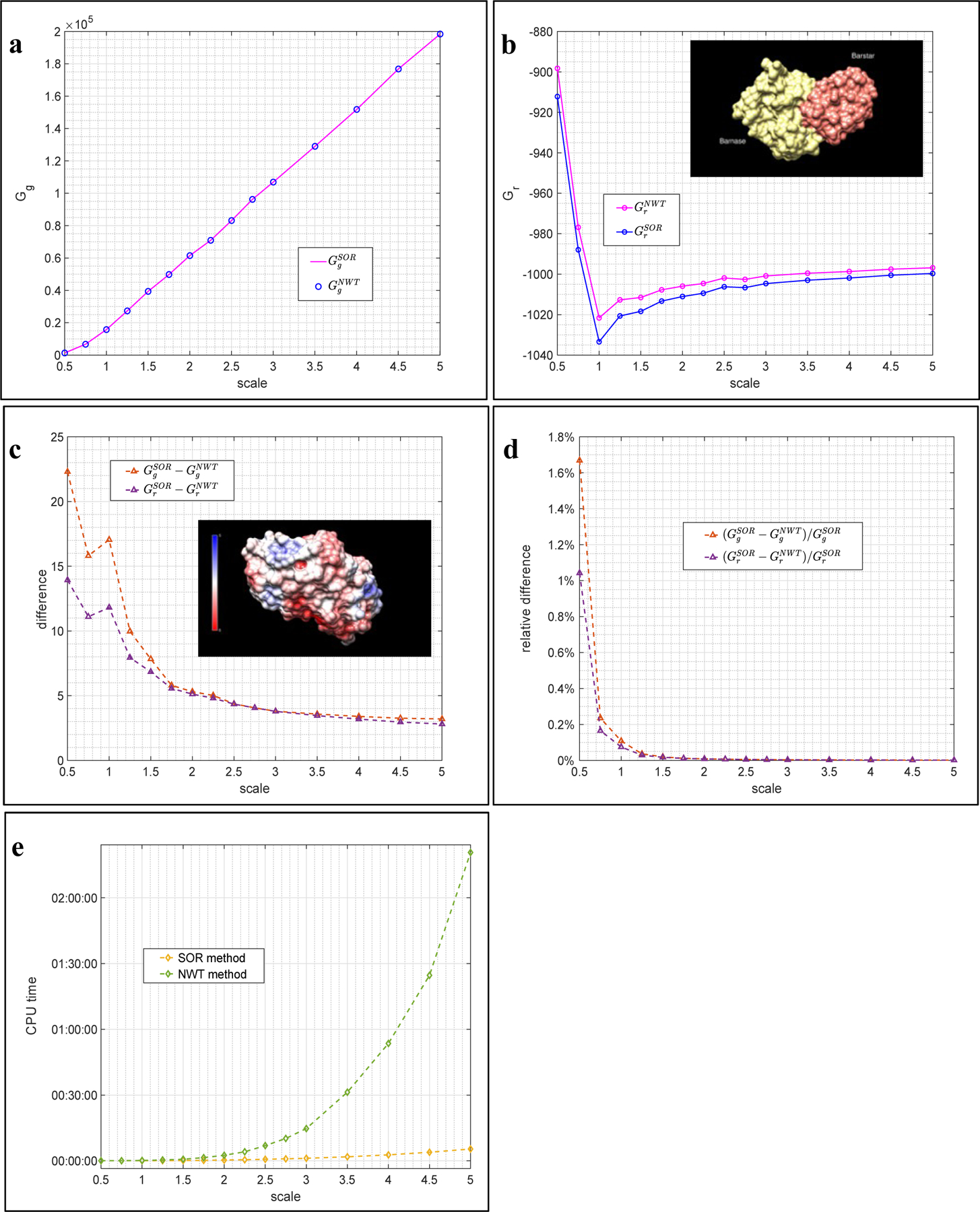
Benchmarks obtained by the SOR and NWT methods with a fixed tolerance TOL = 1.0E-4 and various scales in Example 1. (a) Grid energies. (b) Reaction field energies. (c) Differences. (d) Relative differences. (e) CPU time.

**Example 1.** In the first example, we show that both methods are capable of producing close numerical approximations to the algebraic solution of the discretized NLPBE at a given mesh size *h*. To this end, a basic example of barnase-barstar complex (subfigure in figure 2(b)) is borrowed from DelPhi’s online example repository http://compbio.clemson.edu/delphi and the NLPBE is solved for this complex. Two potential-dependent energies, the total grid energy $G_{g}$ and the corrected reaction field (RXN) energy $G_{r}$, are used to compare the accuracy of the two methods.

The first series of benchmarks is conducted to show that both methods produce closer approximations as the mesh size *h* diminishes. In DelPhi the mesh size *h* is controlled by a parameter *scale*, defined to be the number of grids per angstrom. The mesh size *h* decreases as the scale increases. In this series of benchmarks, the tolerance is fixed, TOL = 1.0E-4, and the scale is varied from scale = 0.5 (29 grids per direction) to scale = 5.0 (293 grids per direction). Energies obtained by the SOR and NWT methods are denoted by $G_{g}^{SOR}$, $G_{r}^{SOR}$, $G_{g}^{NWT}$ and $G_{r}^{NWT}$, respectively. Results are shown in Figure 2.

Obtained energies are shown in Figure 2(a) – (b) first. In both subfigures, it is noticed that energies obtained by the SOR method are always slightly larger than their comparative partners obtained by the NWT method at all tested scales. It shall be pointed out that it may not be the case for other molecules and proteins. It could just be caused by, for instance, the parameter values used in the tests, the initial values used for the iterations, and other factors. Nevertheless, close energies obtained by the two methods at all tested scales evidently demonstrate that they are converging to the exact energies. More detailed comparisons were performed and reported in Figure 2(c) – (d). In these subfigures, the differences, defined as $G_{g}^{SOR}-G_{g}^{NWT}$ and so on, and the relative differences, defined as $\left(G_{g}^{SOR}-G_{g}^{NWT}\right)/G_{g}^{SOR}\times 100\%$ and so on, are shown. The differences, except those at a low scale = 1.0, are seen to approach to a value as small as <5 KT as the scale increases (Figure 2(c)), while the relative differences, starting with an already low percentage ≈1.7%, consistently converge to zero as the scale increases (Figure 2(d)).

In the light that both methods acquire close approximations at all tested scales, and the approximations are getting closer as the scale increases, we conclude that both methods are obtaining close approximations to the same algebraic solutions of the NLPBE at all tested mesh sizes in this example.

Corresponding execution time of the DelPhi program is demonstrated in Figure 2(e) to compare the efficiency of these two methods. One can see that the SOR method costs less time, and therefore is more efficient, at all tested scales. It is primarily due to two reasons. First, the SOR method starts off the nonlinear iterations with better initial values achieved by solving the LPBE for a few dozens of iterations. This numerical treatment significantly reduces the numbers of more costly nonlinear iterations. On the other hand, the NWT method merely uses the default initial values (zeros on all grids) without additional treatments. Secondly, by reusing the saved denominator, each iteration of the SOR method is computationally cheaper than that of the NWT method. As a consequence, the SOR method is found to be more computationally efficient than the NWT method for solving the NLPBE in this example, and it is believed to be the case for many other problems as well.

**Figure 3.**
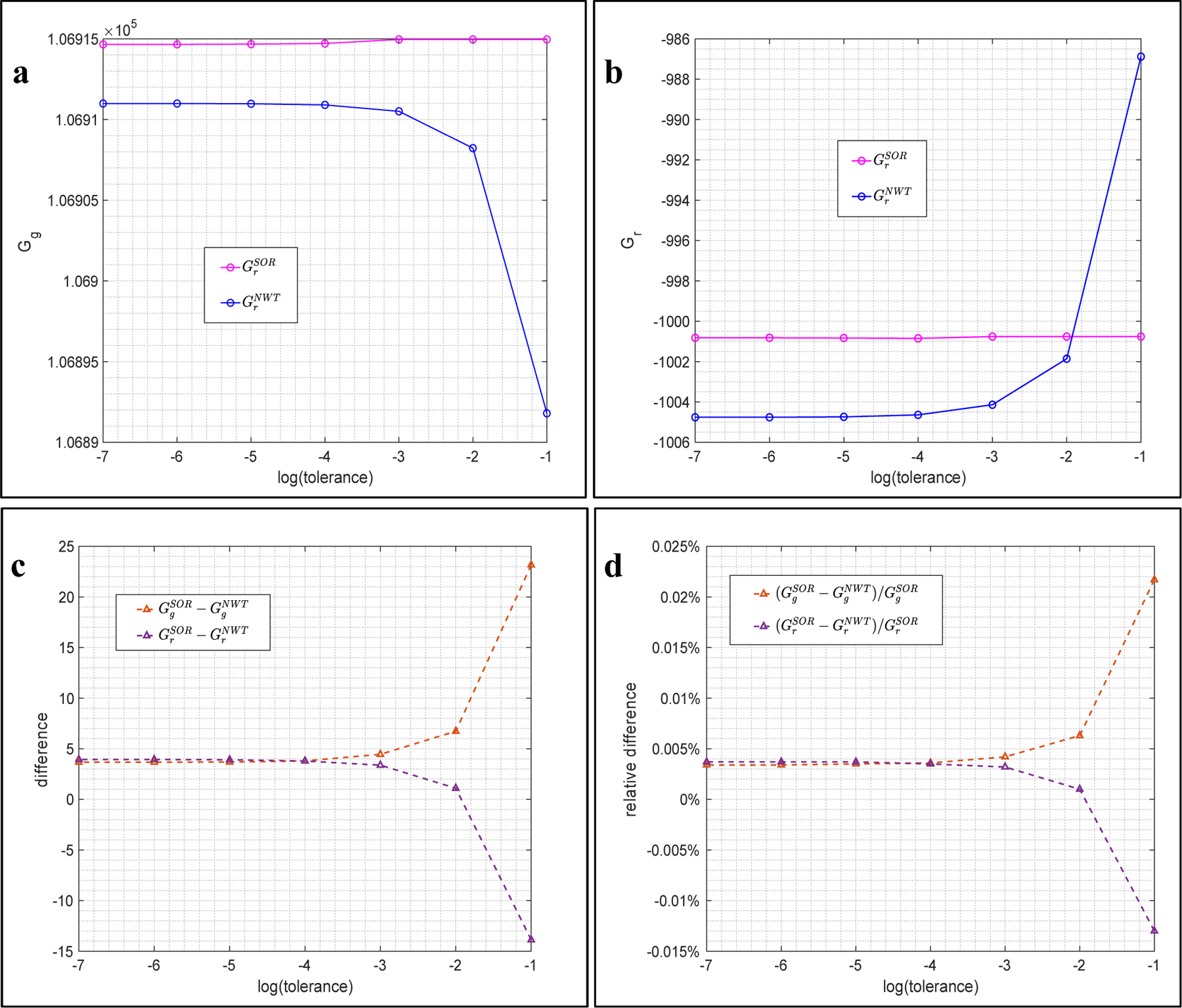
Benchmarks obtained by the SOR and NWT methods with a fixed scale = 2.0 and various tolerances in Example 1. (a) Grid energies. (b) Reaction field energies. (c) Differences. (d) Relative differences.

It has been shown that both methods are capable of achieving close approximations at all scales. We are also interested to see that how fast, in terms of the number of iterations, these two methods can achieve their best approximations at a given scale. To this end, the second series of benchmarks is performed by fixing the scale = 3.0 (175 grids per direction) and varying the tolerance from 1.0E-1 to 1.0E-7. It is naturally expected that both methods will take more iterations, and therefore produce more accurate energies, when smaller tolerance is used. The results shown in Figure 3 match our expectation. Semi-log plots (the horizontal axis is the logarithm of the tolerance) are used in Figure 3 that the scale decreases from the right to left.

Energies, $G_{g}$ and $G_{r}$, obtained by the two methods are presented in Figure 3(a) – (b). One can see that the SOR method shows its stunning efficiency in this example. The obtained curves for the SOR method (pink curves) are almost flat in both subfigures, implying that the SOR method achieves its best approximations without requiring many iterations. In contrast, the NWT method (blue curves) behaves differently: it takes less iterations and achieves coarser approximations when the tolerance is large, and it takes more iterations and obtains finer approximations as the tolerance is smaller. The observed different convergent trends of these two methods can be explained using eqn. (17) – (18). The SOR method tends to add a “big” correction in each iteration to pull $\phi_{0}^{n+1}$ into the range of its best approximation as quickly as possible, so that it does not take too many iterations to attain its best approximation despite which tolerance is actually used. On the contrary, the NWT method adds a moderate correction in each iteration so that it takes more iterations to attain its best, and the approximations are observed to gradually approach to the best as the tolerance decreases. Another important observation on Figure 3(a) – (b) is that $G_{r}^{NWT}$ is larger than $G_{r}^{SOR}$ at tolerance = 1.0E-1 in Figure 3(b). Thus, it is not true that the NWT method always obtains smaller energies.

Differences and relative differences are presented in Figure 3(c) – (d). In both subfigures, all differences and relative differences are found to converge as the tolerance decreases. At the smallest tolerance = 1.0E-7, the differences of $G_{g}$ and $G_{r}$ are found to be as close as <5 KT in Figure 3(c), and the relative differences are found to be as close as <0.005% in Figure 3(d). CPU times of this series of benchmarks are omitted because they are consistent to what shown in Figure 2(e) – the SOR method is more time consuming than the SOR method in all tested cases.

**Example 2.** Results obtained in the first example have provided some insights on the performance of the two methods. We continue to study these two methods for a blindly selected group of proteins. This group of proteins is composed of 15 dimers, and each of them consists of two monomers, namely monomer A and B. More energies, in addition to $G_{g}$ and $G_{r}$, returned by DelPhi will be reported in this example. In particular, they will be used to calculate the binding energy, denoted by ΔG(bind), in this example. Two approaches were suggested in the work[34] to calculate the binding energy. The first approach (approach 1) calculates the electrostatic component of the binding energy from the total nonlinear grid energies of the complex, monomer A and B by

(19) $${\text{ΔG}}_{1}\left(\text{bind}\right)=G_{g}\left(\text{complex}\right)-G_{g}\left(\text{A}\right)-G_{g}\left(\text{B}\right),$$

and the second approach (approach 2) calculates the binding energy from partitioned energies by

(20) $${\text{ΔG}}_{2}\left(\text{bind}\right)=\text{Δ}G_{r}+\text{Δ}G_{\rho }+\text{Δ}G_{o}+\text{Δ}G_{i}+\text{Δ}G_{c}.$$

where $G_{g}$ and $G_{r}$ again denote the total grid energy and the corrected reaction field energy, respectively, $G_{\rho }$ denotes the $\frac{\rho }{\phi }*2$ term in solution, $G_{0}$ denotes the osmotic pressure term, $G_{i}$ denotes the direct ionic contribution inside the box, $G_{c}$ denotes the Coulombic energy, and $\text{Δ}G_{■}$ with the subscript ■ =r,ρ,o,i,c denotes corresponding partitioned energy similar to that defined in eqn. (19). Even though ${\text{ΔG}}_{1}\left(\text{bind}\right)$ and ${\text{ΔG}}_{2}\left(\text{bind}\right)$ are both used to approximate the exact binding energy $\text{ΔG}\left(\text{bind}\right)$, it has been pointed out in the work[34] that they are actually slightly different due to the fact that approach 1 does not fully cancel “artificial grid energy” arising from real charges partitioning onto the grids. Thus, ${\text{ΔG}}_{1}\left(\text{bind}\right)$ is always slightly larger than ${\text{ΔG}}_{2}\left(\text{bind}\right)$. Approach 2 via the energy partition technique does not have such issue so that it is recommended over approach 1.

**Figure 4.**
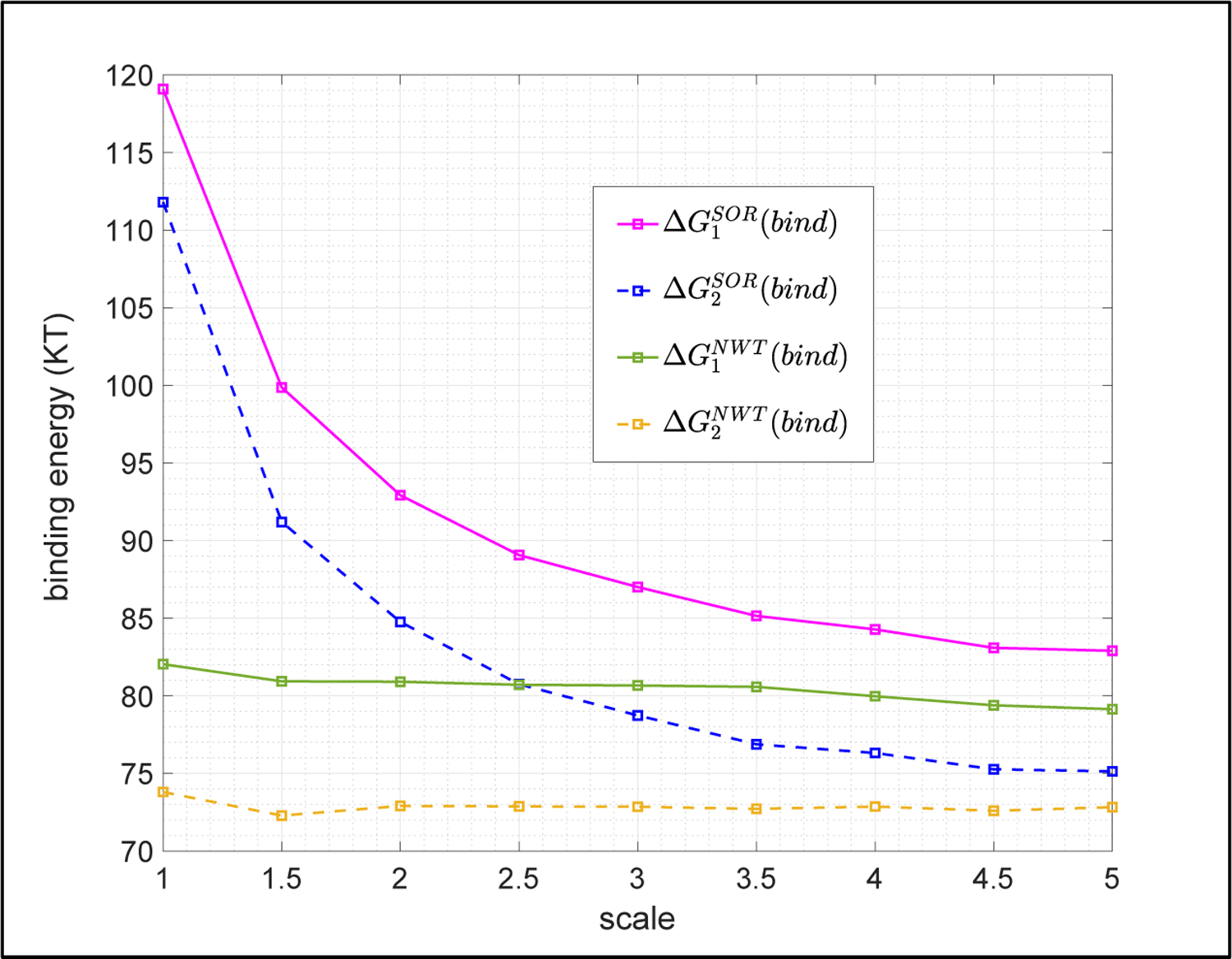
Binding energies obtained by solving the NLPBE via the SOR and NWT methods with a fixed tolerance TOL = 1.0E-4 and various scales for dimer *1fle* in Example 2.

We first show that binding energies calculated via those returned by DelPhi in solving the NLPBE via the SOR and NWT methods are close. To this end, binding energies calculated by both approaches are demonstrated for one dimer, *1fle*. The NLPBE is solved by the SOR and NWT methods with a fixed tolerance = 1.0E-4 and various scales. Calculated binding energies are denoted by ${\text{ΔG}}_{1}^{\text{SOR}}\left(\text{bind}\right)$, ${\text{ΔG}}_{2}^{\text{SOR}}\left(\text{bind}\right)$, ${\text{ΔG}}_{1}^{\text{NWT}}\left(\text{bind}\right)$, and ${\text{ΔG}}_{2}^{\text{NWT}}\left(\text{bind}\right)$, respectively, and demonstrated in Figure 4. A couple of observations can be made on Figure 4. First of all, the two binding energies have the same trend as those obtained by the SOR method that ${\text{ΔG}}_{1}^{\text{NWT}}\left(\text{bind}\right)$ (solid green curve) is always slightly larger than ${\text{ΔG}}_{2}^{\text{NWT}}\left(\text{bind}\right)$ (dashed brown curve) at all tested scales. It matches the statements in the work[34]. Secondly, one can see that ${\text{ΔG}}_{1}^{\text{SOR}}\left(\text{bind}\right)$ and ${\text{ΔG}}_{1}^{\text{NWT}}\left(\text{bind}\right)$ (two solid curves) converge to ${\text{ΔG}}_{1}\left(\text{bind}\right)$, while ${\text{ΔG}}_{2}^{\text{SOR}}\left(\text{bind}\right)$ and ${\text{ΔG}}_{2}^{\text{NWT}}\left(\text{bind}\right)$ (two dashed curves) converge to ${\text{ΔG}}_{2}\left(\text{bind}\right)$, as the scale increases. It is also interesting to point out another important observation, which is not shown in Figure 4. In the benchmarks of dimer *1fle*, we observed that the SOR method is faster than the NWT method in most tested cases. However, there are a few cases in which the NWT method uses the default ω=1.0 and converges without any issue, while the SOR method needs a smaller relaxation parameter, ω=0.5, in order to converge. When it occurs, the SOR method takes significantly more iterations and becomes slower than the NWT method.

**Figure 5.**
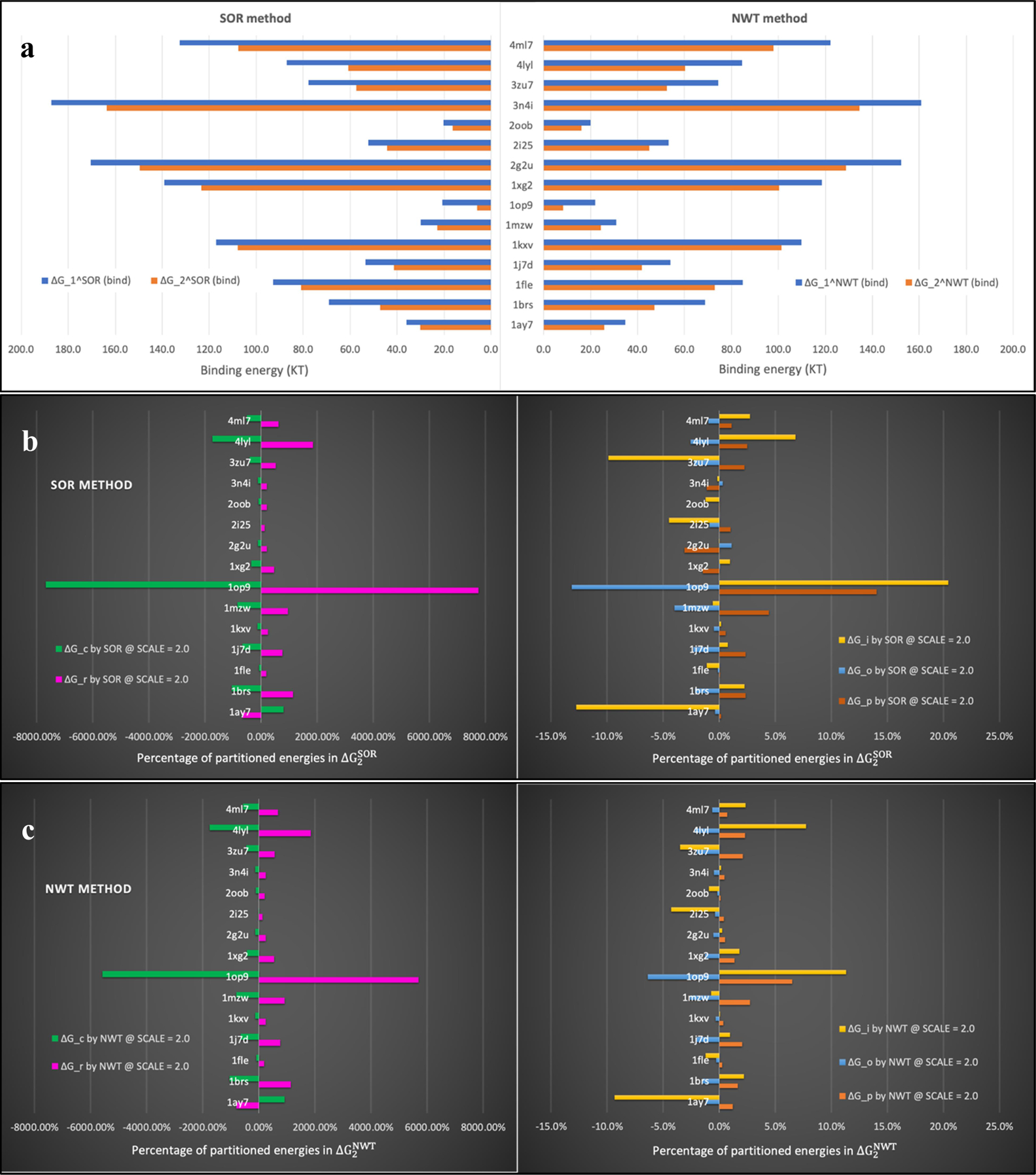
Binding energies and partitioned energies obtained on 15 dimes in Example 2. (a) Binding energies obtained by the SOR method (left panel) and the NWT method (right panel). (b) Percentages of partitioned energies in the binding energy ${\text{ΔG}}_{2}^{\text{SOR}}\left(\text{bind}\right)$. (c) Percentages of partitioned energies in the binding energy ${\text{ΔG}}_{2}^{\text{NWT}}\left(\text{bind}\right)$. Partitioned energies with large magnitudes are shown on the left panel and the remaining energies are shown on the right panel in Figure 5(b) – (c).

The next series of benchmarks was performed to calculate the binding energies at a fixed scale = 2.0 (the most commonly used scale in practice) for all 15 dimers. Results are presented in Figure 5.

In Figure 5(a), the SOR-generated binding energies (${\text{ΔG}}_{1}^{\text{SOR}}\left(\text{bind}\right)$ and ${\text{ΔG}}_{2}^{\text{SOR}}\left(\text{bind}\right)$) are demonstrated on the left panel, and the NWT-generated binding energies (${\text{ΔG}}_{1}^{\text{NWT}}\left(\text{bind}\right)$ and ${\text{ΔG}}_{2}^{\text{NWT}}\left(\text{bind}\right)$) are demonstrated on the right panel. By comparing each blue bar to its paired orange bar on both panels, one can see that the binding energies obtained by approach 1 are always larger than those obtained by approach 2 for all 15 proteins. It is the case for both SOR and NWT methods. Next, by comparing bars in the same color for each dimer on the left and right panels, one can see visible differences on the SOR- and NWT- generated binding energies. However, given the experiences achieved for dimer *1fle*, it is reasonable to expect that these differences are going to diminish if a larger scale is used.

We are interested in seeing how much each individual partitioned energy contributes in the calculated binding energies. Taking ${\text{ΔG}}_{2}\left(\text{bind}\right)$ calculated by eqn. (20) in approach 2 as an example, the percentages of partitioned energies in the binding energy, defined as $\text{Δ}G_{■}/{\text{ΔG}}_{2}\left(\text{bind}\right)\times 100\%$, are shown in 5(b) for the SOR method, and Figure 5(c) for the NWT method, respectively. Percentages of two partitioned energies, $\text{Δ}G_{r}$ and $\text{Δ}G_{c}$, are found to be significantly larger than those of other partitioned energies. Therefore, they are presented on the left panel and others are presented on the right panel in both Figure 5(b) – (c). In these subfigures, one can see that $\text{Δ}G_{r}$ and $\text{Δ}G_{c}$ are always in opposite signs for all 15 dimers, and their sum, $\text{Δ}G_{r}+\text{Δ}G_{c}$, contributes more than 90% of ${\text{ΔG}}_{2}\left(\text{bind}\right)$, while the sum of the remaining three, $\text{Δ}G_{\rho }+\text{Δ}G_{o}+\text{Δ}G_{i}$, contributes less than 10% of ${\text{ΔG}}_{2}\left(\text{bind}\right)$, for all 15 dimers. Moreover, by comparing corresponding energies, it is easy to see that the two methods, SOR and NWT, not only produce similar binding energy ${\text{ΔG}}_{2}\left(\text{bind}\right)$ as a sum of 5 partitioned energies, but also produce similar individual partitioned energy. These partitioned energies, except the partitioned Coulombic energy $\text{Δ}G_{c}$, all depend on the potentials calculated via the SOR and NWT methods. It suggests that the two methods indeed produce close potentials for all 15 dimers.

Above experiments at scale = 2.0 were repeated at a doubled scale, scale = 4.0, and the differences shown in Figure (5) are found to be consistently smaller for all 15 dimers. It evidently shows that one can confidently relies on the energies produced by DelPhi using either method when the iteration process converges at the end. Moreover, we have observed more cases in which the SOR method requires smaller relaxation parameter to converge, while the NWT method has no such issue at all, in the cases tested at scale = 4.0. It inspires us to perform more tests to examine the stability of the two methods.

**Example 3.** It has been observed in Example 2 that the SOR method may require smaller relaxation parameter in order to successfully converge in some cases, while the NWT method never has such issue. Out of abundance of caution, a “crashing” example is purposely created and examined to numerically verify that the NWT method is still able to converge even in some rare and extreme scenarios before we claim that the NWT is a strongly stable method for solving the NLPBE.

This example was tested with a fixed tolerance, TOL = 1.0E-4, and numerous scales ranging from 1.0 to 5.0. This example is believed to be “bizarre” that the iteration process of the SOR method can never be terminated by meeting the desired tolerance at all tested scales. The iteration process is hindered only after a few iterations when the differences of calculated potentials in two successive iterations are large at some grids, causing the SOR method relentlessly seek for smaller relaxation parameter ω to reduce these differences before moving forward to the next iteration. This effort repeats many times in each of the first several iterations and prevents the iterations progress properly towards the end. As a consequence, the SOR method fails to produce any energies in this example after waiting for a long time.

**Figure 6.**
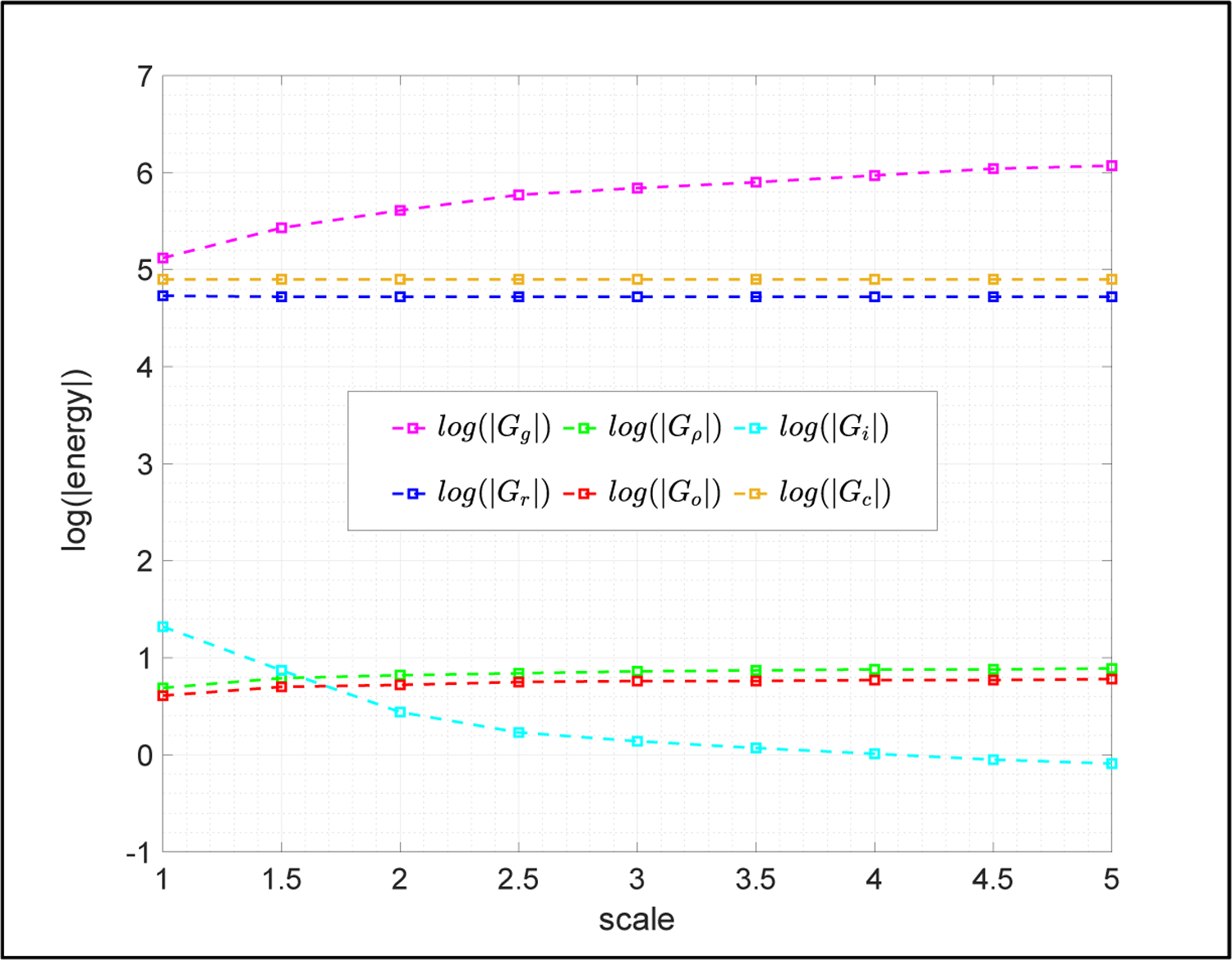
DelPhi returned energies obtained by solving the NLPBE via the NWT method with a fixed tolerance = 1.0E-4 in Example 3.

It is a completely different story for the NWT method. The NWT method merely uses the default ω=1.0 and converges successfully in all tested cases. Energies produced by DelPhi running the NWT method are presented by a semi-log plot (the vertical axis is the logarithms of the absolute values of the energies) in Figure 6. One can see that all energies behave normally without any unanticipated outcomes.

Additional examples beside Example 3 have been tested as well and we have not seen one case that the NWT method fails to converge. The experiences we earned make us confidently claim that the newly developed NWT method is a reliable alternative to solve the NLPBE for problems with high nonlinearity. Meanwhile, bearing in mind that the SOR method is still more efficient in many cases, the SOR method is still recommended to solve the LPBE/NLPBE when no divergence issue takes place. In the cases that the SOR method has troubles to converge, one can immediately observe in DelPhi’s outputs that the iteration stops progressing forward, the relaxation parameter becomes smaller, and the calculated tolerances get larger. It will be enough to tell that the SOR method is having troubles to converge, and it is advised to stop the program and switch to the NWT method.

## 4. Discussion and Conclusion

In this work, a newly developed Newton-like method is proposed. It has been implemented in the DelPhi program to solve the PBE for electrostatic potentials. It has been demonstrated that the NWT method is relatively slower, equally accurate, and more stable compared to the SOR method for solving the NLPBE. The merits of the new NWT method make it a valuable add-on to the DelPhi program. The NWT method is recommended to the computational molecular society to solve the NLPBE for problems with strong nonlinearity when other solvers have trouble to converge and deliver reliable solutions. Developments to improve the efficiency of the NWT method will be carried out and reported in the future.

## References

[1] Cisneros GA, Karttunen M, Ren P, , Classical electrostatics for biomolecular simulations, Chemical reviews, 114 (2014), 779–814. 23981057 10.1021/cr300461d PMC3947274

[2] Honig B and Nicholls A, Classical electrostatics in biology and chemistry, Science, 268 (1995), 1144–1149. 7761829 10.1126/science.7761829

[3] Zhang Z, Witham S and Alexov E, On the role of electrostatics in protein–protein interactions, Physical biology, 8 (2011), 035001. 21572182 10.1088/1478-3975/8/3/035001 PMC3137121

[4] Batra J, Szabó A, Caulfield TR, , Long-range electrostatic complementarity governs substrate recognition by human chymotrypsin C, a key regulator of digestive enzyme activation, Journal of Biological Chemistry, 288 (2013), 9848–9859. 23430245 10.1074/jbc.M113.457382 PMC3617285

[5] Ikeuchi H, Ahn Y-M, Otokawa T, , A sulfoximine-based inhibitor of human asparagine synthetase kills L-asparaginase-resistant leukemia cells, Bioorganic & medicinal chemistry, 20 (2012), 5915–5927. 22951255 10.1016/j.bmc.2012.07.047

[6] Huang X, Dong F and Zhou H-X, Electrostatic recognition and induced fit in the κ-PVIIA toxin binding to Shaker potassium channel, Journal of the American Chemical Society, 127 (2005), 6836–6849. 15869307 10.1021/ja042641q

[7] Alexov E, Numerical calculations of the pH of maximal protein stability: The effect of the sequence composition and three‐dimensional structure, European Journal of Biochemistry, 271 (2004), 173–185. 14686930 10.1046/j.1432-1033.2003.03917.x

[8] Isvoran A, Craescu C and Alexov E, Electrostatic control of the overall shape of calmodulin: numerical calculations, European Biophysics Journal, 36 (2007), 225–237. 17285296 10.1007/s00249-006-0123-1

[9] Mitra RC, Zhang Z and Alexov E, In silico modeling of pH‐optimum of protein–protein binding, Proteins: Structure, Function, and Bioinformatics, 79 (2011), 925–936. 10.1002/prot.22931 PMC3213863 21287623

[10] Onufriev AV and Alexov E, Protonation and pK changes in protein–ligand binding, Quarterly reviews of biophysics, 46 (2013), 181–209. 23889892 10.1017/S0033583513000024 PMC4437766

[11] Talley K and Alexov E, On the pH‐optimum of activity and stability of proteins, Proteins: Structure, Function, and Bioinformatics, 78 (2010), 2699–2706. 10.1002/prot.22786 PMC2911520 20589630

[12] Petukh M, Kimmet T and Alexov E, BION web server: predicting non-specifically bound surface ions, Bioinformatics, 29 (2013), 805–806. 23380591 10.1093/bioinformatics/btt032 PMC3597141

[13] Petukh M, Zhang M and Alexov E, Statistical investigation of surface bound ions and further development of BION server to include p H and salt dependence, Journal of computational chemistry, 36 (2015), 2381–2393. 26484964 10.1002/jcc.24218

[14] Petukh M, Zhenirovskyy M, Li C, , Predicting nonspecific ion binding using DelPhi, Biophysical journal, 102 (2012), 2885–2893. 22735539 10.1016/j.bpj.2012.05.013 PMC3379622

[15] Alexov E, Mehler EL, Baker N, , Progress in the prediction of pKa values in proteins, Proteins: Structure, Function, and Bioinformatics, 79 (2011), 3260–3275. 10.1002/prot.23189 PMC3243943 22002859

[16] Georgescu RE, Alexov EG and Gunner MR, Combining conformational flexibility and continuum electrostatics for calculating pKas in proteins, Biophysical journal, 83 (2002), 1731–1748. 12324397 10.1016/S0006-3495(02)73940-4 PMC1302268

[17] Gunner MR and Baker NA, Continuum electrostatics approaches to calculating pKas and Ems in proteins. Methods in enzymology: Elsevier (2016), pp. 1–20. 10.1016/bs.mie.2016.05.052 PMC5380367 27497160

[18] Bertonati C, Honig B and Alexov E, Poisson-Boltzmann calculations of nonspecific salt effects on protein-protein binding free energies, Biophysical journal, 92 (2007), 1891–1899. 17208980 10.1529/biophysj.106.092122 PMC1861767

[19] Bredenberg JH, Russo C and Fenley MO, Salt-mediated electrostatics in the association of TATA binding proteins to DNA: a combined molecular mechanics/Poisson-Boltzmann study, Biophysical journal, 94 (2008), 4634–4645. 18326635 10.1529/biophysj.107.125609 PMC2397334

[20] Ghosh A, Rapp CS and Friesner RA, Generalized Born model based on a surface integral formulation, The Journal of Physical Chemistry B, 102 (1998), 10983–10990.

[21] Grochowski P and Trylska J, Continuum molecular electrostatics, salt effects, and counterion binding—a review of the Poisson–Boltzmann theory and its modifications, Biopolymers: Original Research on Biomolecules, 89 (2008), 93–113. 10.1002/bip.20877 17969016

[22] Baker NA, Poisson–Boltzmann methods for biomolecular electrostatics. Methods in enzymology: Elsevier (2004), pp. 94–118. 10.1016/S0076-6879(04)83005-2 15063648

[23] Xiao L, Diao J, Greene DA, , A continuum Poisson–Boltzmann model for membrane channel proteins, Journal of chemical theory and computation, 13 (2017), 3398–3412. 28564540 10.1021/acs.jctc.7b00382 PMC5728381

[24] Li C, Li L, Petukh M, , Progress in developing Poisson-Boltzmann equation solvers, Computational and Mathematical Biophysics, 1 (2013), 42–62. 10.2478/mlbmb-2013-0002 PMC3816640 24199185

[25] Mongan J, Simmerling C, McCammon JA, , Generalized Born model with a simple, robust molecular volume correction, Journal of chemical theory and computation, 3 (2007), 156–169. 21072141 10.1021/ct600085e PMC2975579

[26] Feig M, Onufriev A, Lee MS, , Performance comparison of generalized born and Poisson methods in the calculation of electrostatic solvation energies for protein structures, Journal of computational chemistry, 25 (2004), 265–284. 14648625 10.1002/jcc.10378

[27] Li L, Li C, Sarkar S, , DelPhi: a comprehensive suite for DelPhi software and associated resources, BMC biophysics, 5 (2012), 9. 22583952 10.1186/2046-1682-5-9 PMC3463482

[28] Rocchia W, Sridharan S, Nicholls A, , Rapid grid‐based construction of the molecular surface and the use of induced surface charge to calculate reaction field energies: Applications to the molecular systems and geometric objects, Journal of computational chemistry, 23 (2002), 128–137. 11913378 10.1002/jcc.1161

[29] Botello-Smith WM, Liu X, Cai Q, , Numerical Poisson–Boltzmann model for continuum membrane systems, Chemical physics letters, 555 (2013), 274–281. 23439886 10.1016/j.cplett.2012.10.081 PMC3579545

[30] Zhou Y, Zhao S, Feig M, , High order matched interface and boundary method for elliptic equations with discontinuous coefficients and singular sources, Journal of Computational Physics, 213 (2006), 1–30.

[31] Jurrus E, Engel D, Star K, , Improvements to the APBS biomolecular solvation software suite, Protein Science, 27 (2018), 112–128. 28836357 10.1002/pro.3280 PMC5734301

[32] Boschitsch AH and Fenley MO, A new outer boundary formulation and energy corrections for the nonlinear Poisson–Boltzmann equation, Journal of computational chemistry, 28 (2007), 909–921. 17238171 10.1002/jcc.20565

[33] Boschitsch AH and Fenley MO, Hybrid boundary element and finite difference method for solving the nonlinear Poisson–Boltzmann equation, Journal of computational chemistry, 25 (2004), 935–955. 15027106 10.1002/jcc.20000

[34] Li C, Jia Z, Chakravorty A, , DelPhi Suite: New Developments and Review of Functionalities, Journal of Computational Chemistry, 40 (2019), 2502–2508. 31237360 10.1002/jcc.26006 PMC6771749

[35] Klapper I, Hagstrom R, Fine R, , Focusing of electric fields in the active site of Cu‐Zn superoxide dismutase: Effects of ionic strength and amino‐acid modification, Proteins: Structure, Function, and Bioinformatics, 1 (1986), 47–59. 10.1002/prot.340010109 3449851

[36] Im W, Beglov D and Roux B, Continuum solvation model: computation of electrostatic forces from numerical solutions to the Poisson-Boltzmann equation, Computer physics communications, 111 (1998), 59–75.

[37] Rocchia W, Alexov E and Honig B, Extending the applicability of the nonlinear Poisson− Boltzmann equation: multiple dielectric constants and multivalent ions, The Journal of Physical Chemistry B, 105 (2001), 6507–6514.

[38] Luty BA, Davis ME and McCammon JA, Solving the finite‐difference non‐linear Poisson–Boltzmann equation, Journal of computational chemistry, 13 (1992), 1114–1118.

[39] Holst MJ and Saied F, Numerical solution of the nonlinear Poisson–Boltzmann equation: developing more robust and efficient methods, Journal of computational chemistry, 16 (1995), 337–364.

[40] Cai Q, Hsieh M-J, Wang J, , Performance of nonlinear finite-difference Poisson− Boltzmann solvers, Journal of Chemical Theory and Computation, 6 (2010), 203–211. 24723843 10.1021/ct900381r PMC3979552

[41] Shestakov A, Milovich J and Noy A, Solution of the nonlinear Poisson–Boltzmann equation using pseudo-transient continuation and the finite element method, Journal of colloid and interface science, 247 (2002), 62–79. 16290441 10.1006/jcis.2001.8033

[42] Sayyed–Ahmad A, Tuncay K and Ortoleva PJ, Efficient solution technique for solving the Poisson–Boltzmann equation, Journal of computational chemistry, 25 (2004), 1068–1074. 15067682 10.1002/jcc.20039

[43] Geng W and Zhao S, Fully implicit ADI schemes for solving the nonlinear Poisson-Boltzmann equation, Computational and Mathematical Biophysics, 1 (2013), 109–123.

[44] Nicholls A and Honig B, A rapid finite difference algorithm, utilizing successive over‐relaxation to solve the Poisson–Boltzmann equation, Journal of computational chemistry, 12 (1991), 435–445.

